# SKILL BUILDING BLOCKS: ASYNCHRONOUS ONLINE LEARNING THROUGH INTERACTIVE THEMATIC INSTRUCTION

**DOI:** 10.1093/geroni/igac059.1717

**Published:** 2022-12-20

**Authors:** Harvey Sterns, Vicki de Klerk-Rubin

**Affiliations:** The University of Akron, Akron, Ohio, United States; Validation Training Institute, Pleasant Hill, Oregon, United States

## Abstract

Whether professional or as a family carer, interactions with an older adult living with or without cognitive decline should be person-centered. Learning behavioral skills requires more than watching a demonstration or cognitive learning; it requires practice, feedback and more practice. Online learning has become the best way to reach carers of older adults, partially due to the COVID pandemic and partially due to time, money and energy resources being stretched to their limits. The Validation Training Institute has developed 6 self-directed, online Skill Building Blocks that uses thematic instruction strategy, interactive exercises, and videos demonstrating why each skill is important and how to best utilize the skill in real life situations. Learners have completed pre and post-questionnaires using the Likert scale and the IMMS (Instructional Material Motivation Survey) which measures attention, relevance, confidence, and satisfaction. An online focus group of participants concluded the data collection process. Techniques taught through Skill Building Blocks proved to be relevant to the learners’ life experience. There was effective retention and transfer of skills, and the training proved to be motivating for continued application in the real world. Learners expressed confidence and satisfaction with the user-friendly, thematic design of each block.

